# Social, Psychological, and Environmental-Structural Factors Associated with Tobacco Experimentation among Adolescents in Shanghai, China 

**DOI:** 10.3390/ijerph9103421

**Published:** 2012-09-26

**Authors:** Yong Cai, Lin Lu, Na Li, Jingfen Zhu, Yaping He, Pamela Redmon, Abhinav Goyal, Cheng Huang, Yun Qiao, Jin Ma

**Affiliations:** 1 School of Public Health affiliated with School of Medicine, Shanghai Jiaotong University, Shanghai 200025, China; Email: caiyong@shsmu.edu.cn (Y.C.); lulin-618@163.com (L.L.); lina_shsmu@yahoo.com.cn (N.L.); zhujingfenjt@163.com (J.Z.); hypcyr@hotmail.com (Y.H.); 2 Global Health Institute, Emory University, Atlanta, GA 30322, USA; Email: predmon@emory.edu (P.R.); agoyal4@emory.edu (A.G.); chenghuang@gwu.edu (C.H); 3 Pudong Institute for Health Development, Shanghai 200129, China; Email: joannajun@163.com

**Keywords:** tobacco experimentation, adolescent, environmental-structural factors, associations

## Abstract

*Objective*: To estimate the prevalence and social, psychological and environmental-structural determinants of tobacco experimentation among adolescents in Shanghai, China. *Methods*: We conducted a cross-sectional study based on a two-stage cluster sample design by using the Chinese version of the Global Youth Tobacco Survey (GYTS) to investigate smoking behavior among 19,117 students from 41 junior and senior high schools in Shanghai, China. The association between potential factors and tobacco experimentation were assessed using complex samples procedure logistic regression. Results: Of the 19,117 respondents, 10.5% (15.3% boys and 6.2% girls) reported the tobacco experimentation. The main social, psychological, and environmental-structural factors associated with tobacco experimentation were having close friends who smoke (AOR = 8.21; 95% CI: 6.49–10.39); one or both parents smoking (AOR 1.57; CI: 1.39–1.77); a poor school tobacco control environment (AOR 1.53; CI: 1.37–1.83); a high acceptance level of tobacco use (AOR 1.44; CI: 1.28–1.82); and a high level of media tobacco exposure (AOR 1.23; CI: 1.10–1.37). Peer smoking might contribute to smoking experimentation among girls (AOR 8.93; CI: 5.84–13.66) more so than among boys (AOR 7.79; CI: 5.97–9.94) and media tobacco exposure had no association with tobacco experimentation among female students. *Conclusions*: Social, psychological, and environmental factors are closely associated with tobacco experimentation among adolescents. Prevention programs aimed at reducing teen tobacco experimentation should be conducted at home and school with support by parents, peers and teachers. Our findings should prove useful for future development of intervention strategies among adolescents in Shanghai, China.

## 1. Introduction

Tobacco is a leading cause of preventable morbidity and mortality globally and it exerts a substantial negative impact on people’s health [1,2]. The World Health Organization (WHO) estimates that approximately over 1 billion people smoke tobacco currently, among whom 12% of adolescent boys and 7% of adolescent girls smoke cigarettes [3,4]. Over 80% of smokers live in low- and middle-income countries [5]. The tobacco epidemic in China is also responsible for an enormous burden of disease and poses a significant challenge to the country’s public health system [6]. According to nationally representative data from the Global Adult Tobacco Survey (GATS) conducted during 2008–2010, the estimated prevalence of current tobacco use among adults aged 15 years and above is 28.1%, which corresponds to nearly 0.38 billion smokers in China in year 2010 [7]. Current estimates are that one million people in China will die annually of diseases related to smoking, and this will increase to two million by 2025 [8]. Studies in developed countries show that most people begin using tobacco in their teens, and assessing tobacco use by youth through the Global Youth Tobacco Survey (GYTS) developed by WHO and the US CDC forms an important part of the global tobacco surveillance system [9,10,11]. Tobacco experimentation (smoking one or two puffs of a cigarette) among young people in China is high: in 1999, about 16%–30% of school-children aged 13–15 in four provinces reported having smoked cigarettes [9]. According to statistics, currently there are about 15 million smokers and 40 million people who are trying to smoke among 130 million adolescents aged 13 to 18 in China [12]. Especially alarming are sharp increases in tobacco use recently documented among Chinese adolescents and that the problem of youth smoking in China is becoming more serious as the age of smoking initiation becomes lower and lower [8]. Smoking is not only harmful to adolescents’ physiological health, but also damages their psychological health [13,14]. Therefore, tobacco use and adolescent smoking have become a public health problem that urgently needs to be solved by reducing the overall smoking rate in the World. The behavior of tobacco experimentation is influenced by personal factors and various external factors as well, including physiological, psychological, social, and environmental factors [15,16,17,18]. Some cross-sectional surveys in China have documented the smoking prevalence and the associated social and psychological factors in early youth [19,20]. However, behavioral change is not only base on individual cognitive theories, but also environmental-structural factors that play an important role in these conceptual frameworks. Environmental-structural factors typically refer to elements outside the control of or the cognition of, individuals, such as social norms, material and human resources, and policies and legislation that facilitate or constrain individual behavior [21]. In order to understand the current situation of tobacco experimentation among adolescents and to discover related influencing factors, we conducted research based on a questionnaire of the WHO Global Youth Tobacco Survey (GYTS). GYTS is a school-based survey of a defined geographic site that can be a country, a province, a city, or any other geographic entity. Our research not only focused on general social and psychological factors but also on environmental-structural factors related to tobacco experimentation among junior and senior high school students in Shanghai. We believe our results will be helpful to formulate policies related to smoking prevention and control among adolescents in the future. 

## 2. Methods

### 2.1. Study Setting

This study was conducted in Shanghai, one of the largest cities in China with a population of 17 to 18 million. 

### 2.2. Sample and Procedure

We conducted a two-stage cluster sample design by using a Chinese version of the Global Youth Tobacco Survey (GYTS) to examine smoking behavior among junior and senior high school students. This self-administered, school-based instrument had been tested in preliminary research and showed suitability by the reliability and validity analysis [22]. The Chinese-version GYTS served as a reference for Chinese communities to plan surveillance and tobacco control programs for teenage smoking behavior [22]. GYTS used a standardized methodology for constructing sampling frames, selecting schools and classes, preparing questionnaires, conducting field procedures, and processing data [23]. Representative students were sampled using a two-stage cluster sample design in our research. In the first stage, three districts of Shanghai (Huangpu, Pudong and Minhang) were randomly selected and the sampling frame included all schools in the three districts. Forty-one junior and senior high schools (including technical schools) were randomly chosen from the three districts determined as the subjects of study. In the second sampling stage, classes within chosen schools were randomly selected and all the students came from seventh-grade to eleventh-grade. However, twelfth-grade students were preparing for the national college entrance examination at the time of sampling; Thus, this group of students was excluded in the sampling frame. All students in selected classes who attended school the day the survey was administered were eligible to participate. The sample size of the field survey was 19,976 students, from whom 19,117 usable questionnaires were collected for a response rate of 95.7%. We compared the age × gender distribution from grade 7 to grade 11 (usually aged from 11.5 to 17) of the sample with the target population (data from the sixth population census of Shanghai) and found no significant difference between them.

### 2.3. Measures

The questionnaire we used was the Chinese version of the Global Youth Tobacco Survey (GYTS) developed by Taipei Medical University in the year 2008 [22]. Trained investigators explained the survey to students, answered their question, and collected the questionnaires. All the students were given time during regular school hours to complete their own self-reported questionnaire forms anonymously.

The Chinese version of GYTS addressed the important tobacco experimentation indicator: “Have you ever tried or experimented with cigarette smoking, even one or two puffs?” as well as a response other than “definitely no” to the questions. The questionnaire also inquired about an individual’s key socio-demographic characteristics (e.g., age, gender, school type, hometown, experience with tobacco experimentation, pocket money each month, education level of parents, and academic performance in the class), predisposing factors of tobacco use (e.g., cognition concerning dangers of tobacco use, acceptance of tobacco use, and exposure to smoking by parents and friends), enabling, and reinforcing factors including media exposure, and environmental-structural aspects of tobacco control. We developed our own scoring methods as follows and which were tested by reliability analysis [24]:

#### 2.3.1. Score of Acceptance of Tobacco Use

As for knowledge and behavior aspects of the survey, 12 questions involving interpersonal relationships, personality charm, image and health hazards were included (Cronbach’s Alpha 0.714). Each item was assigned from 3 points to 1 point. The higher the score, the more easily the advantages of tobacco are accepted. The final score was obtained by adding the total points of the 12 questions. The median was used as an indicator to divide items into different groups. A final score lower than the median, was divided into a low tobacco acceptance group, and a score higher than the median was divided into a high tobacco acceptance group.

#### 2.3.2. Score of Media Exposure Related Tobacco

In terms of media publications concerning tobacco, nine questions are asked (Cronbach’s Alpha 0.693). According to information acquired by exposure to publications, each question was assigned 3 points to 1 point (or 4 points to 1 point). The higher the score, the more information was obtained by exposure to the publications. The final exposure score was obtained by adding the total points of the nine questions. The median was used as an indicator to divide items into different groups. A final score higher than the median was divided into the high media tobacco exposure group, and a score lower than the median was divided into the low media tobacco exposure group.

#### 2.3.3. Score of Environmental-Structural Factors of Tobacco Control

Environmental-structural aspects of tobacco control factors in our study mainly focused on policies and legislation that facilitate or constrain individual behavior. There were 12 items related to smoking control including tobacco control policies of schools, health promotion by tobacco control, and tobacco consumption inside and outside of schools. Environmental-structural factors in every school were evaluated by the 12 items (Cronbach’s Alpha 0.762). The heads of the schools were asked to respond to the 12 items with “yes”, “no” or “do not know”. Positive answers were credited with a score of one, while negative answers or responses of “do not know” received a score of zero. The sum of each question’s score was converted into a total score, with the maximum being 12. Overall score was divided into ranges from “0–4”, “5–8”and “9–12” which respectively represent “poor”, “middle”, “good” environmental-structural factors related to tobacco control in each school.

### 2.4. Data Analysis

Data were double-entered using Epidata 3.0 software. All statistical analyses were performed using the Complex Samples procedure from Statistical Package for Social Sciences (SPSS vision 20.0) for Windows. A weighting factor was applied to each student record to adjust for non response (by school, class and student) and variation in the probability of selection at the school and class levels. By using Complex Samples procedure, we calculated the prevalence (95% confidence intervals) of tobacco experimentation. Unadjusted odd ratios (OR) with associated 95% confidence intervals (CI) were calculated by logistic regression analysis to examine the differences among key individual, social psychological and environmental-structural factors. Multivariate logistic regression with forward stepwise technique was used to examine the association between dichotomous smoking behavior variables and individual, social-psychological and environmental-structural factors simultaneously. The adjusted odds ratios (AOR) with associated 95% confidence intervals (CI) were adjusted for controlling for other possible contributions from other variables in the model.

## 3. Results

### 3.1. Main Characteristics of Individual of the Sample

We obtained the data of the sixth population census in Shanghai from the Statistics Bureau and compared the age*gender distribution from grade 7 to grade 11(usually aged from 11.5 to 17) with the sample and found no significant differences between them. The gender distribution of the youth was balanced, 9,783 (51.2%) were male students and 9,334 (48.8%) were female students. (Table 1). The average age was 13.64 ± 0.95 (range 11–17) years old and 8,050 (42.1%) were aged from 13–15 years. Results are shown in Table 1.

**Table 1:** The age × gender distribution of the sample and target population (thousand).

	Sample	Shanghai (population census of year 2011)
	**Male (%) Female (%) total **	**Male (%) Female (%) total **
<13	1.571 (50.5) 1.541 (49.5) 3.112 (16.3)	110.36 (52.9) 98.44 (47.1) 208.8 (15.4)
13–15	4.019 (49.9) 4.031 (50.1) 8.050 (42.1)	294.04 (50.3) 290.26 (49.7) 584.3 (43.2)
>15	4.193 (52.7) 3.762 (47.3) 7.955 (41.6)	287.85 (51.3) 272.75 (48.7) 560.6 (41.4)
total	9.783 (51.2) 9.334 (48.8) 19.117 (100.0)	692.25 (51.1) 661.45 (48.9) 1,353.7 (100.0)

### 3.2. Association between Individual, Social Psychological, Environmental-Structural Factors and Tobacco Experimentation

As shown in Table 2, 19,117 students participated in the study and over 1 in 10 (10.5%; 95% CI: 9.7%–11.4%) of them reported an experience of tobacco experimentation. The relationship between tobacco experimentation and each individual, social and psychological factor or environmental-structural support factor was examined first. The tobacco experimentation rate was significantly different among the three districts. Students from Pudong were less likely to experiment with tobacco than the other two districts (*P* < 0.001). The rate of tobacco experimentation among male students was 15.3% which was significantly higher than 6.2% among female students (*P* < 0.001). The rate of tobacco experimentation increased significantly from 3.8% to 15.8% as the growth of age increased (*P* < 0.001). Over three in 10 technical students who had the experience of tobacco experimentation was significantly higher than students from junior and senior high school (*P* < 0.001). Students who came from suburb were more likely to try smoking than students from downtown (*P* < 0.05). Participants whose pocket money was more than 400 yuan (about 63 $US) per month were significantly more likely to try tobacco than those with less pocket money (*P* < 0.001). The education level of parents was also significantly associated with rate of tobacco experimentation rate by their children: the lower the education level of parents, the higher the rate of tobacco experimentation children experienced (*P* < 0.001). The rate of tobacco experimentation among adolescents was strongly associated with exposure to smoking by their parents and friends. Students with parents who smoke had a tobacco experimentation rate of 12.7% *versus* 7.6% for those whose parents did not smoke (*P* < 0.001). Students who had friends that smoke experienced a high rate of tobacco experimentation, 55.0%, which was almost eleven-fold high than students whose friends did not smoke, 5.2% (*P* < 0.001).The rate of tobacco experimentation among adolescents with the lowest academic performance in the class, 14.8%, was significantly higher than those with the highest academic performance, 7.8% (*P* < 0.001). Students with high opiniors as to benefits and acceptance of tobacco use were more likely to try tobacco than those with lower opinions, 13.7% *versus* 7.8% (*P* < 0.001). The rate of tobacco experimentation varied significantly between adolescents with the lowest level of environment-structural supporting for tobacco controls in schools, 13.6%, compared with those with the highest levels of support for tobacco controls, 8.2% (*P* < 0.001). Media tobacco exposure also played important roles in tobacco experimentation among youths, students exposed to more information about tobacco use experienced a high rate of tobacco experimentation, 12.6%, compared with students who had less tobacco exposure from media, 8.7% (*P* < 0.001). 

**Table 2:** Association between main characteristics of individual, social psychological, and environmental-structural factors and prevalence of tobacco experimentation among adolescents in Shanghai (N = 19,117).

Variable	Sorts	Count	Prevalence % (95% CI)	Unadjusted OR (95% CI)
Total	-	19,117	10.5 ( 9.7–11.4)	- -
Districts	Pudong	5,909	7.3 ( 6.5– 8.2)	1 -
	Minhang	9,976	11.8 (10.5–13.2)	1.70** (1.43–2.03)
	Huangpu	3,232	12.1 (10.2–14.4)	1.76** (1.39–2.22)
Year of age group	<13	3,112	3.8 (3.0–4.9)	1 -
	13–15	8,050	6.6 (5.8–7.5)	1.78** (1.35–2.34)
	>15	7,955	15.8 ( 14.5–17.3)	4.70** (3.56–6.20)
School type	Junior high school	12,413	6.2 (5.5–6.9)	1 -
	Senior high school	4,375	10.4 ( 9.1–11.8)	1.77** (1.46–2.13)
	Technical school	2,329	30.6 (28.0–33.4)	6.74** (5.66–8.02)
Hometown	Urban	10,480	9.1 (8.3–10.0)	1.35* (1.18–1.56)
	Suburb	8,185	12.0 (10.7–13.3)	
Pocket money/Month(yuan)	<400 (63US$)	15,646	8.7 (8.0–9.4)	1 -
	≥400 (63US$)	3,108	19.6 (17.7–21.6)	2.58** (2.31–2.88)
Education level of father	Primary school or lower	649	19.9 (16.7–23.5)	1 -
	Middle school	4,480	14.2 (12.6–15.9)	0.66**(0.55–0.81)
	Senior high school	6,269	10.3 (9.4–11.4)	0.46**(0.38–0.57)
	Junior college	2,592	9.1 (7.8–10.5)	0.40**(0.31–0.53)
	Undergraduate or higher	4,951	6.9 (6.0–7.9)	0.30**(0.23–0.39)
Education level of mother	Primary school or lower	1,264	20.1 (17.1–23.5)	1 -
	Middle school	5,000	13.6 (12.0–15.3)	0.62**(0.51–0.76)
	Senior high school	5,673	8.9 ( 8.1–9.8)	0.39**(0.32–0.48)
	Junior college	2,870	8.7 ( 7.0–9.6)	0.35**(0.28–0.46)
	Undergraduate or higher	4,181	7.7 ( 6.7–8.9)	0.33**(0.26–0.43)
Smoking or not (friends)	No	14,084	5.2 (4.7–5.7)	1 -
	Partly	45,07	21.5 (19.8–23.2)	4.99** (4.34–5.73)
	Most or all	442	55.0 (50.3–59.7)	22.33** (18.02–27.67)
Academic performance in the class	First 25%	5,390	7.8 (6.9–8.8)	1 -
	Middle	8,238	9.4 (8.5–10.4)	1.23* (1.07–1.41)
	Last 25%	5,228	14.8 (13.5–16.2)	2.05** (1.78–2.35)
Environmental-structural score	High (9–12)	6,152	8.2 (7.3–9.2)	1 -
	Middle (5–8)	5,960	9.6 ( 8.6–10.6)	1.19* (1.03–1.37)
	Low (0–4)	6,631	13.6 (12.4–14.7)	1.76** (1.57–1.98)
Media tobacco exposure score	Low (0–10)	9,913	8.7 (7.8–9.6)	1 -
	High (11–22)	8,547	12.6 (11.6–13.6)	1.52* (1.37–1.68)
Tobacco acceptance score	Low (0–17)	10,615	7.8 (7.1–8.6)	1 -
	High (18–36)	7,849	13.7 (12.7–14.9)	1.88* (1.70–2.07)

OR, odds ratio; CI, confidence interval *P* value: * <0.05; ** <0.001.

### 3.3. Determinants of Tobacco Experimentation among Adolescents in Shanghai China using a Multivariate Logistic Regression Model

As shown in Table 3, the relationship between tobacco experimentation and individual, social-psychological or environmental-structural support factors was examined by a multivariate forward stepwise logistic regression model. It was statistically significant with twelve variables remained determining the tobacco experimentation. Male students were more likely to try tobacco than female students (adjusted odds ratio (AOR) 2.05; 95% confidence interval (CI) 1.78–2.35). Students from the Minhang and Huangpu district were significantly more likely than students from the Pudong district to have ever smoked cigarettes (AOR 1.55 and 1.44; CI: 1.32–1.82 and 1.15–1.82). Older students (>15 years and 13–15 years) were more likely to use tobacco than the youngest (AOR 2.08 and 1.41; CI: 1.48–2.93 and 1.07–1.87). Technical students were more likely to try tobacco than junior high school students (AOR 2.03; CI: 1.57–2.61). Students whose parents smoke were significantly more likely to use tobacco (AOR 1.53; CI: 1.36–1.73). Students who had friends that almost all smoke had an over 8-fold increase in the odds of smoking compared to those who had no friends who smoke (AOR 8.29; CI: 6.49–10.39). Participants were more likely to try tobacco if they had much more pocket money/month (AOR 1.57; CI: 1.39–1.77). The higher the education level of the father got, the lower the rate of tobacco experimentation by his children (AOR 0.84; CI: 0.79–0.90). Adolescents with the lowest academic performance in the class held strong acceptance attitudes toward tobacco use (AOR 1.39; CI: 1.19–1.63). The rate of tobacco experimentation varied significantly between students learning in schools with the lowest levels of environment-structural support for tobacco control when compared with those with the highest levels of environment-structural support (AOR 1.53; CI: 1.37–1.83). Students exposed to much more information about tobacco consumed from media experienced higher rates of tobacco use than those with less exposure (AOR 1.23; CI: 1.10–1.37). Students with higher opinions concerning the benefits and acceptance of tobacco use were more likely to try tobacco than those with lower opinions (AOR 1.44; CI: 1.28–1.82). 

**Table 3:** Determinants of tobacco experimentation among adolescents in Shanghai China by using a multivariate logistic regression model (N = 19,017).

Variable		AOR	95% CI
District: Minhang (reference group: Pudong)		1.55**	1.32–1.82
Huangpu (reference group: Pudong)		1.44**	1.15–1.82
Gender: male (reference group: female)		2.05**	1.78–2.35
Year of age: 13–15 (reference group: age < 13)		1.41**	1.07–1.87
>15 (reference group: age < 13)		2.08**	1.48–2.93
School type: Senior high school (reference group: junior high school)		0.86	0.66–1.13
Technical school(reference group: junior high school)		2.03**	1.57–2.61
Pocket money: ≥400 (63US$)/month (reference group: <400)		1.48**	1.31–1.68
Parent smoke: yes (reference group: no)		1.57**	1.39–1.77
Friends smoke: Partly yes (reference group: no)		2.66**	2.30–3.08
Most yes (reference group: no)		8.21**	6.49–10.39
Education level of father:		0.84*	0.79–0.90
Academic performance: Middle (reference group: first 25%)		1.09	0.94–1.28
	Last 25% (reference group: first 25%)	1.39*	1.19–1.63
Environmental-structural score: Middle (reference group: high)		1.05	0.90–1.24
	Low (reference group: high)	1.53*	1.37–1.83
Media tobacco exposure score: High (reference group: low)		1.23*	1.10–1.37
Tobacco acceptance score: High (reference group: low)		1.44**	1.28–1.82

AOR, adjusted odds ratio; CI, confidence interval *P* value: * <0.05; ** <0.001.

### 3.4. Variables Associated with Tobacco Experimentation among Adolescents of Different Gender in Shanghai China

As shown in Table 4, there were some different variables related to tobacco experimentation when we separate the regression models for boys and girls. It was still statistically significant with eleven variables remained determining the tobacco experimentation among male students which was the same as among all students. However, the variables reduced to nine when we presented separate regression models for female students. It appeared that academic performance and media tobacco exposure had no association with tobacco experimentation among girls. We also found that the peer smoking contributed to tobacco experimentation among girls (AOR 8.93; CI: 5. 84–13.66) more so than among boys (AOR 7.59; CI: 5.79–9.94).

## 4. Discussion

The prevalence of tobacco experimentation was 10.5% among in-school adolescents aged 11–17 years in Shanghai (China) as revealed by the Chinese version of the GYTS questionnaire. A cross country comparison (within the GYTS project) reported that tobacco experimentation rates varied from 12.1% in Sri Lanka to 73.6% in Ukraine (Kiev) among students aged 13–15 years (median: 24.2%) [9]. The wide variation could be attributed to factors like different smoking control strategies, cultural, religious norms and availability of tobacco products. Our present study indicated that the tobacco experimentation rate is 6.6% among the target age group from 13–15 years which was significantly lower than the rate of 16%–30% twelve years ago from four Chinese provinces’ GYTS [9]. Qiu Xin *et al.* performed a similar investigation among junior high school students in Hangzhou (a city locating very close to Shanghai) and found the prevalence of tobacco experimentation was 9.7% [19]. Johnson *et al.* found that smoking varied across cities, with a higher smoking prevalence in southwestern cities and a lower prevalence in coastal cities of China [25]. We speculate that the four provinces were mainly located in the West or North of China while Shanghai and Hangzhou are Eastern coastal cities of China, which may explain the different rates of tobacco experimentation among youth. Another explanation is that for the past 10 years, a vigorous campaign called “action on smoke-free school” has been implemented by Shanghai Municipal Education Commission, which may have reduced the tobacco experimentation rate by informing adolescents of the harmful effects of smoking. It seems that adolescents in Shanghai have maintained a low prevalence of tobacco experimentation in the recent years, reflected by the 6.9% rate among junior high school students reported by Yao *et al.* in 2008 [20]. The WHO requested that countries repeat the GYTS every three years for 13–15 year old students from junior high school [3] and we suggested that such requests should be extended to include students from 16–18 year old from senior high schools as well.

**Table 4:** Variables associated with tobacco experimentation among adolescents of different gender by using a multivariate logistic regression model.

Variable		AOR (95% CI )	
		Male students	Female students
District: Minhang (reference group: Pudong)		1.64** (1.35–2.00)	1.43** (1.10–1.85)
Huangpu (reference group: Pudong)		1.57** (1.21–2.05)	1.24 (0.86–1.78)
Year of age: 13–15 (reference group: age < 13)		1.30 (0.93–1.81)	1.62** (1.01–2.59)
>15 (reference group: age < 13)		2.12** (1.41–3.19)	1.88** (1.02–3.46)
School type: Senior high school (reference group: junior high school)		0.84 (0.60–1.18)	1.00 (0.62–1.63)
Technical school (reference group: junior high school)		2.11** (1.41–3.19)	1.98** (1.24–3.16)
Pocket money: ≥400 (63US$)/month (reference group: <400)		1.56** (1.32–1.83)	1.36* (1.07–1.71)
Parent smoke: yes (reference group: no)		1.52** (1.31–1.76)	1.70** (1.36–2.13)
Friends smoke: Partly yes (reference group: no)		2.35** (1.95–2.84)	3.35** (2.64–4.23)
Most yes (reference group: no)		7.59** (5.79–9.94)	8.93** (5.84–13.66)
Education level of father:		0.86** (0.81–0.92)	0.90* (0.83–0.98)
Academic performance: Middle (reference group: first 25%)		1.16 (0.96–1.42)	-
	Last 25% (reference group: first 25%)	1.60** (1.30–1.97)	-
Environmental-structural score: Middle (reference group: high)		0.88 (0.72–1.05)	1.17 (0.93–1.36)
	Low (reference group: high)	1.48** (1.39–1.82)	1.55** (1.17–1.99)
Media tobacco exposure score: High (reference group: low)		1.32* (1.19–1.50)	-
Tobacco acceptance score: High (reference group: low)		1.41** (1.21–1.64)	1.47** (1.27–1.75)

AOR, adjusted odds ratio; CI, confidence interval *P* value: * <0.05; ** <0.001.

In multivariate analysis districts, school type, age, gender, pocket money, education level of the father, parents and peers smoking status, academic performance, tobacco control environment of school, media exposure and attitude toward tobacco use were significant determinants of tobacco experimentation in youths. We found it notable that students from the Minhang and Huangpu districts were more likely to try tobacco than students from the Pudong district. This can probably be attributed to special smoking control strategies, including tobacco control preventions conducted in many schools of Pudong (a pilot district) in Shanghai recently. The tobacco experimentation rate of students from technical schools was higher than those from junior and senior high schools. This can probably be attributed to weak actions concerning smoke-free schools in many technical schools and the relatively poorer academic performance among these students, because we found that adolescents with the lowest academic performance held strong acceptance of tobacco use especially among boys. This finding was similar with the results of Bergen *et al.* who reported that perceived academic performance was associated with tobacco use among adolescents [26]. We found that receiving pocket money was associated with smoking, as had also been reported by Siziya *et al.* [27] in Kaful (Zambia). We believe that having much more disposable cash may increase the chance of adolescents spending the money to buy cigarettes since they usually are not refused purchase from stores in China. It was not surprising that age, gender, parental and peer smoking, and attitudes toward tobacco use have been associated with tobacco experimentation based on the social ecological model [28]. Peer smoking tended to be the most important influencing factors, and similar causes have been proposed in many studies at home and abroad [16,17,18,20]. There is little doubt that having a friend who smokes was a major contributor to an individual’s experimenting with tobacco. However, it was interesting that girls were more vulnerable to be influenced by peer smoking than boys when we separated the multivariate logistic regression models based on gender. The risk of tobacco experimentation of those students whose parents (father, mother or both) smoke was 1.57 times higher than those students whose parents did not smoke. Parental smoking may influence youth to start smoking, as had been reported elsewhere [29,30]. We applied the GYTS questionnaire to analyze the degree of acceptance for smoking. The risk of tobacco experimentation of those students who think smoking can improve interpersonal relationships, can present a positive image to others, and has little negative effect on health is about 1.5 times higher than those with the opposite viewpoints. This result signifies the adverse effects of peer smokers, and cultural factors also exert a substantial impact on adolescent tobacco experimentation. We also evaluated environment-structural support for tobacco control including the household’s and school’s tobacco control environment. Poor environment-structural support for tobacco control was also the main risk factor for smoking initiation among youth and tobacco control strategies should be focused not only on community settings but also school and family settings. Last but not least, median tobacco exposure such as cigarette advertisements on billboards, newspapers, TV programs, magazines and sports activities also contributed to smoking initiation among students, especially boys. This issue will require substantial attention from the decision makers and policy makers in providing necessary regulation in particularly for controlling any form of the cigarette advertisement. 

Among the strengths of our research was the fact that the sample was large, allowing for precise effects to be estimated. However, the present study had several limitations. First, the participants selected came from Shanghai, one of the largest metropolitan areas in China, and may not reflect the cultural diversity of rural areas. Second, all twelfth-grade students were excluded from the survey, which might limit generalization of the research finding. Third, data were based on the self-reporting by students, who might misreport their behaviors or attitudes. While this bias cannot be determined from the data, reliability and validity studies had indicated good test-retest and good fit of the results for the Chinese version of the GYTS questionnaire we used [22,24]. 

In 2011, Shanghai passed the latest tobacco control policy which banned smoking in any public places. Unfortunately, while the law was strong, the compliance with the law has been low because the enforcement of its provisions was weak and the penalties are not severe enough. However, Shanghai needs to develop and implement effective enforcement strategies now since the tobacco epidemic in China is responsible for an enormous burden of disease and poses a significant challenge to the country’s public health system [6]. Since most smokers began smoking in their adolescence and it is difficult to quit smoking cigarettes [31], the period of junior and senior high school is of great importance to begin early intervention related to tobacco use. Both psychological and environmental factors should be taken into consideration for promoting health by tobacco control. Family and school environments are the most important elements to achieve effective tobacco control. The findings from our study have direct policy implications and should contribute to the design of evidence-based measures to prevent smoking among adolescents in Shanghai China. We suggested that additional research is needed to evaluate the effectiveness of current tobacco control interventions for youth.

## 5. Conclusions

We found that the prevalence of tobacco experimentation was 10.5% among in-school adolescents in Shanghai, which was lower than the rate observed when the GYTS was conducted twelve years ago in four cities of China. This reduction might be attributed to the popularization of tobacco control and health promotion policies such as “action on smoke free school” in Shanghai in recently years. Individual, social, and psychological determinants such as gender, age, pocket money, parents and peer smoking status, academic performance, media tobacco exposure and attitude toward tobacco use play important roles in tobacco initiation among youth. Girls were more vulnerable to influence by peer smoking and boys were sensitive to media exposure by tobacco advertisements. We also confirmed the association between the environment-structural support for tobacco control and tobacco experimentation among in-school adolescents. We suggested it is necessary to provide regulation for controlling any form of cigarette advertising. Prevention programs aimed at reduce teen tobacco experimentation should be conducted at home and school and supported by parents, peers, and teachers. 
